# Spore morphology and its systematic implication in *Pteris* (Pteridaceae)

**DOI:** 10.1371/journal.pone.0207712

**Published:** 2018-11-26

**Authors:** Yi-Shan Chao, Yao-Moan Huang

**Affiliations:** 1 Department of Biomedical Science and Environmental Biology, Kaohsiung Medical University, Kaohsiung, Taiwan; 2 Division of Silviculture, Taiwan Forestry Research Institute, Taipei, Taiwan; University of North Carolina at Greensboro, UNITED STATES

## Abstract

*Pteris* (Pteridaceae) spores are usually trilete and can be distinguished by the perine ornamentation. The systematic value of spore morphology in *Pteris* is unclear, especially based on the renewed infrageneric classification of *Pteris*. In the present study, we used scanning electron microscopy (SEM) to understand spore characters in 57 *Pteris* species, one *Onychium* species, and two *Astrolepis* species; 40 species are reported here for the first time. The observed spore characters combined with published spore data, totaling 100 species from 16 sections of *Pteris*, were mapped onto a reconstructed phylogenetic tree. Seven characters (five proposed in previous studies), including an equatorial flange, laesural ridges, proximal ridges, distal ridges, tubercula on distal faces, coarse reticula on distal faces, and a row of extervermiculi between the distal face and equatorial flange, were analyzed to investigate spore morphology evolution in *Pteris*. However, the results showed no synapomorphies with other genera in Pteridaceae. Most of the characters were found to have arisen independently several times in different lineages or were even frequently reversed. Equatorial flanges and tubercula on distal faces are plesiomorphies and present in most *Pteris* species. Overall, the application of spore morphology in section circumscription is limited. Thus, we suggest combining spore morphology with leaf characters for *Pteris* infrageneric classification.

## Introduction

Phylogenetic studies of the genus *Pteris* (Pteridaceae) have revealed its major lineages. The monophyly of the genus *Pteris* has been confirmed using six cpDNA and one nuclear markers, and *P*. *platyzomopsis* Christenh. & H. Schneid. (*Platyzoma microphyllum* R. Br.) is included in this genus [1]. Furthermore, *Pteris* is classified into three subgenera and 16 sections [2]. However, most leaf characters were shown to have arisen several times in different lineages or were even frequently reversed [3]. Homoplasy results show that the leaf morphological characters cannot circumscribe sections well, even when combined with the geographic distribution.

Spore morphology has been applied for species delimitation, especially for ferns with similar leaf morphologies, such as *Pteris decrescens* Christ and *P*. *parviloba* Christ [4], the *Elaphoglossum ciliatum* (C. Presl) T. Moore group [5], and *Adiantopsis* species (Pteridaceae) with palmately compound laminae [6]. It was also demonstrated that spore morphology is useful to determine relationships among taxa to higher than species level, such as genus delimitation under Adiantaceae [7]; characterize bolbitidoid genera (Dryopteridaceae) [8]; and infer transformation series for spore types of *Lomariopsis* (Lomariopsidaceae) [9].

Previous studies have tried to evaluate taxonomic information on the spore morphology of *Pteris*, based on perine ornamentation. Tryon & Lugardon [10] determined that most *Pteris* spores are trilete with an equatorial flange. To our knowledge, this is the first study to classify *Pteris* spore morphology based on the geographic distributions of 18 *Pteris* species. Dai et al. [11] generalized the spore morphology of 30 *Pteris* species and four varieties in China; *Pteris* spores are classified as four groups: (1) without an equatorial flange; (2) with an equatorial flange but without proximal and distal ridges; (3) with an equatorial flange, proximal ridge, and distal ridge; and (4) with an equatorial flange and a row of extervermiculi between the distal face and equatorial flange. The results are incongruent with the previously circumscribed infrageneric classification in China: sections *Campteria*, *Quadriauricula*, and *Pteris* [12]. Yang et al. [13] examined 40 species and varieties of *Pteris* in China. They used characters similar to those used by Dai et al. [11], but classified the spores into six types and thought that most species could have several different types. Based on the studies of 25 *Pteris* species in Mexico and Mesoamerica, Palacios-Rios et al. [14] proposed that the commissural flange (laesural ridges) and ornamentation in *Pteris* spores were of taxonomic value. Ornamentation, such as tuberculate and reticulate patterns, had not been applied for spore classification in previous studies. These were only used for the description of specific species. In general, previous studies are limited by specific geographic areas or small species sample size. The systematic values of *Pteris* spore morphologies, especially based on the revised phylogenetic classification, have not been studied. The evolution pattern of spore morphology remains unclear.

Using scanning electron microscopy (SEM), this study was undertaken to investigate the spore morphology of the genus *Pteris*. To infer the systematics values of the spore characters, we reconstructed a phylogeny based on DNA sequences from previous studies and then mapped spore characters. Here, considering previous spore studies, an overview of worldwide *Pteris* spore evolution is provided. The taxonomic value of spore morphologies in infrageneric delimitation is discussed.

## Materials and methods

### Phylogenetic analyses

A phylogenetic tree based on the cpDNA dataset, *rbcL* and *matK*, was constructed. The sequences of 180 *Pteris* species and 21 outgroup species from other genera of Pteridaceae were collected (S1 Table). One hundred thirty species have both *rbc*L and *mat*K sequences, most from Chao et al. [3]; 50 species have only *rbc*L sequences from GenBank. *Acrostichum aureum* L. and *Ceratopteris thalictroides* (L.) Brongn. comprised the most phylogenetically distant outgroups [15].

Alignment was performed with ClustalW [16] as implemented in BioEdit v.7.0.5 [17], manually checked, and revised where necessary. Gaps were treated as missing characters. The resulting sequence data matrices were analyzed to infer phylogenetic relationships, using Bayesian inference. For the combination of *rbc*L and *mat*K markers, the best-fitting evolutionary model for each partition was GTR+I+Γ [18] under the Akaike Information Criterion [19] in Modeltest v.3.7 [20].

*Pteris* phylogeny was inferred by Bayesian inference (BI) was conducted using MrBayes v.3.2.1 [21, 22]. Ten million generations of four Markov chain Monte Carlo (MCMC) were run. Two independent runs were conducted to avoid getting stuck upon local optima; the temperature of the heated chains was set to 0.2. Trees were sampled every 1,000 generations, and the first 1,000 trees were discarded as burn-in. Convergence was checked by Tracer 1.6 [23]. Then, Bayesian posterior probabilities (BPP) were determined from the 50% majority-rule consensus tree of the retained trees.

### Spore morphologies

In order to analysis all sections of *Pteris*, we examined the spores of as many species as possible, including 57 *Pteris* species, one *Onychium* species, and two *Astrolepis* species (60 species). For each species, one or two specimens and 30–100 spores were examined. Most of the sampled spores were taken from the DNA vouchers cited by previous samples for phylogenetic studies. Other sampled spores were from another specimen with fertile leaves because the cited DNA vouchers were unavailable or had no fertile leaves. Voucher information is provided in S1 Table. To observe detailed spore morphologies, spores were air-dried, scattered, and then mounted onto aluminum scanning electron microscope stubs. The stubs were then coated with gold in a sputter coater for 3 min. Samples were visualized via SEM; the accelerating voltage was 15 kV (TM3000; Hitachi, Tokyo, Japan). The proximal and distal views of each species were examined and recorded. The morphological terminology of spores followed Tryon & Lugardon [10] and Huang [24].

### Ancestral character state reconstruction

To infer the systematics values of the spore characters, the key characters applied to the classification of *Pteris* spores in previous studies were investigated [10,11,13,14]. A distal ridge means a distinct prominent platform on distal face, parallel to an equatorial flange. Three laesurae are usually flanked by conspicuous ridges (laesural ridges). Because ornamentation of spores is different on proximal ridges and distal faces and most diverse on distal faces, tubercles and coarse reticula were also recorded. The various characters were defined as absent or present as follows: (1) an equatorial flange, (2) laesural ridges, (3) proximal ridges (peripheral ridges paralleled to equatorial flange), (4) distal ridges, (5) tubercula on distal faces, (6) coarse reticula on distal faces, and (7) a row of extervermiculi between the distal face and equatorial flange. Some intermediate characters were coded as ambiguous.

To reconstruct the ancestral character states of spore morphologies, previous research about *Pteris* morphologies from SEM were consulted [4,10,11,13,14,25–27]. Sixty species from this study and 40 species from other studies described the spore characters of a total of 100 species, which cover all sections of *Pteris*, except section *Dentatae*. These multiple characters were summarized to build a character matrix containing the seven morphological characters.

Character tracing was performed using the most parsimonious reconstruction of ancestral states in Mesquite v3.40 [28]. Characters were treated as unordered, categorical variables and mapped onto the phylogenetic trees obtained from the cpDNA dataset to infer patterns of evolution.

## Results

### *Pteris* phylogeny

**Fig 1:**
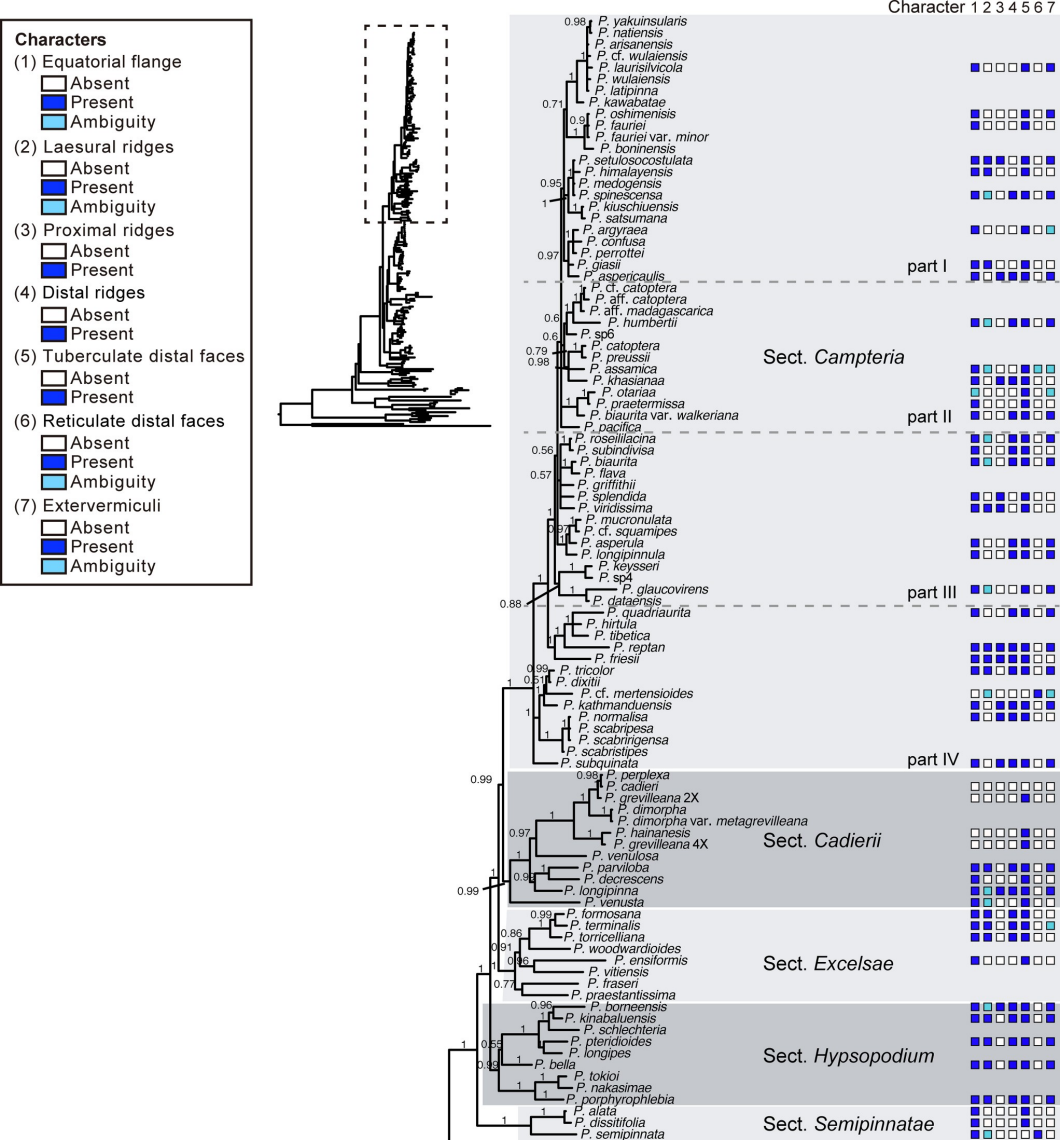
Phylogeny of *Pteris*, with spore morphology, was inferred from Bayesian inference. Values above branches show BI posterior probabilities. States are indicated for the following characters: (1) an equatorial flange, (2) laesural ridges, (3) proximal ridges, (4) distal ridges, (5) tubercula on distal faces, (6) coarse reticula on distal faces, and (7) a row of extervermiculi between the distal face and equatorial flange.

**Fig 2:**
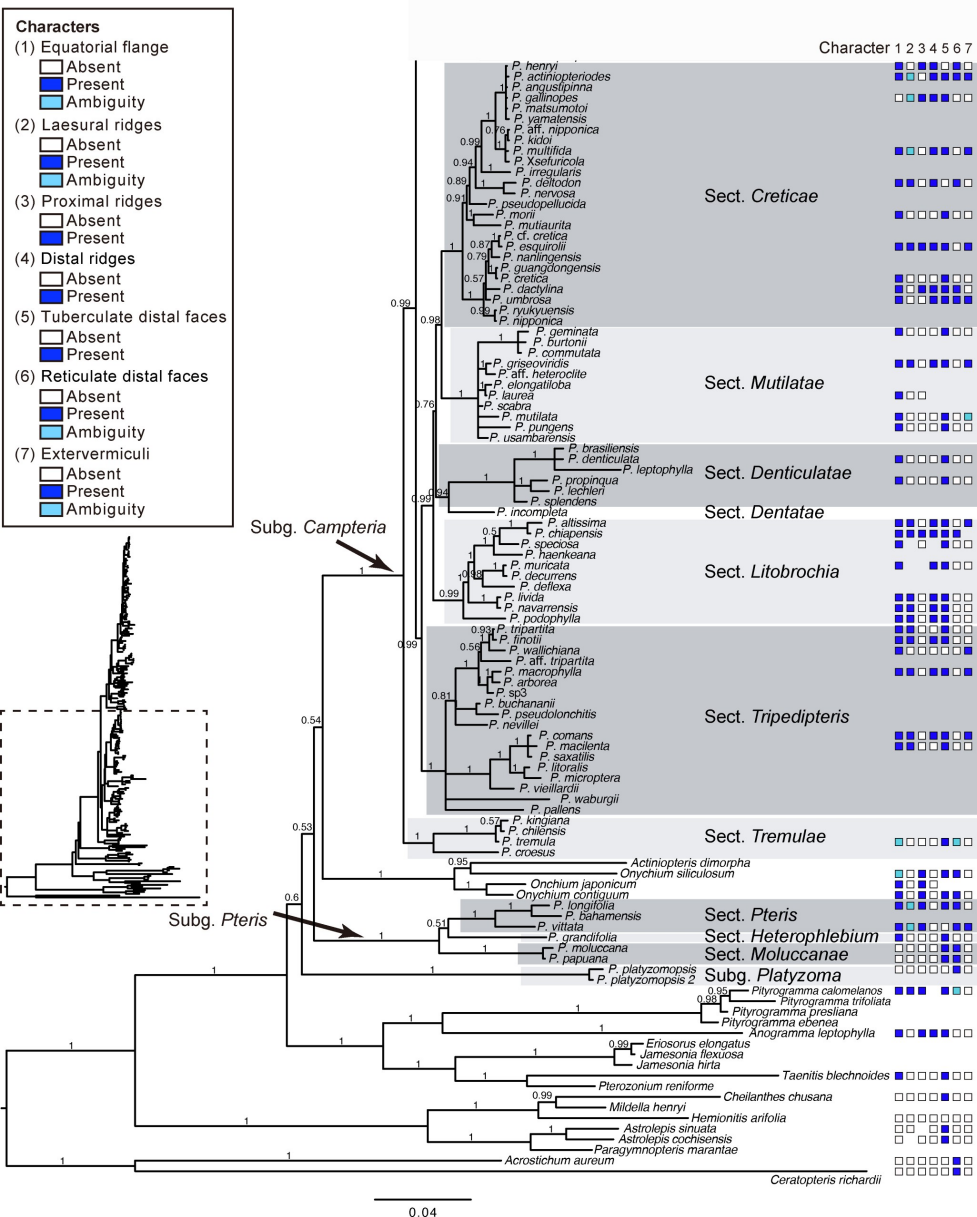
Phylogeny of *Pteris*, with spore morphology, was inferred from Bayesian inference (continued).

**Fig 3:**
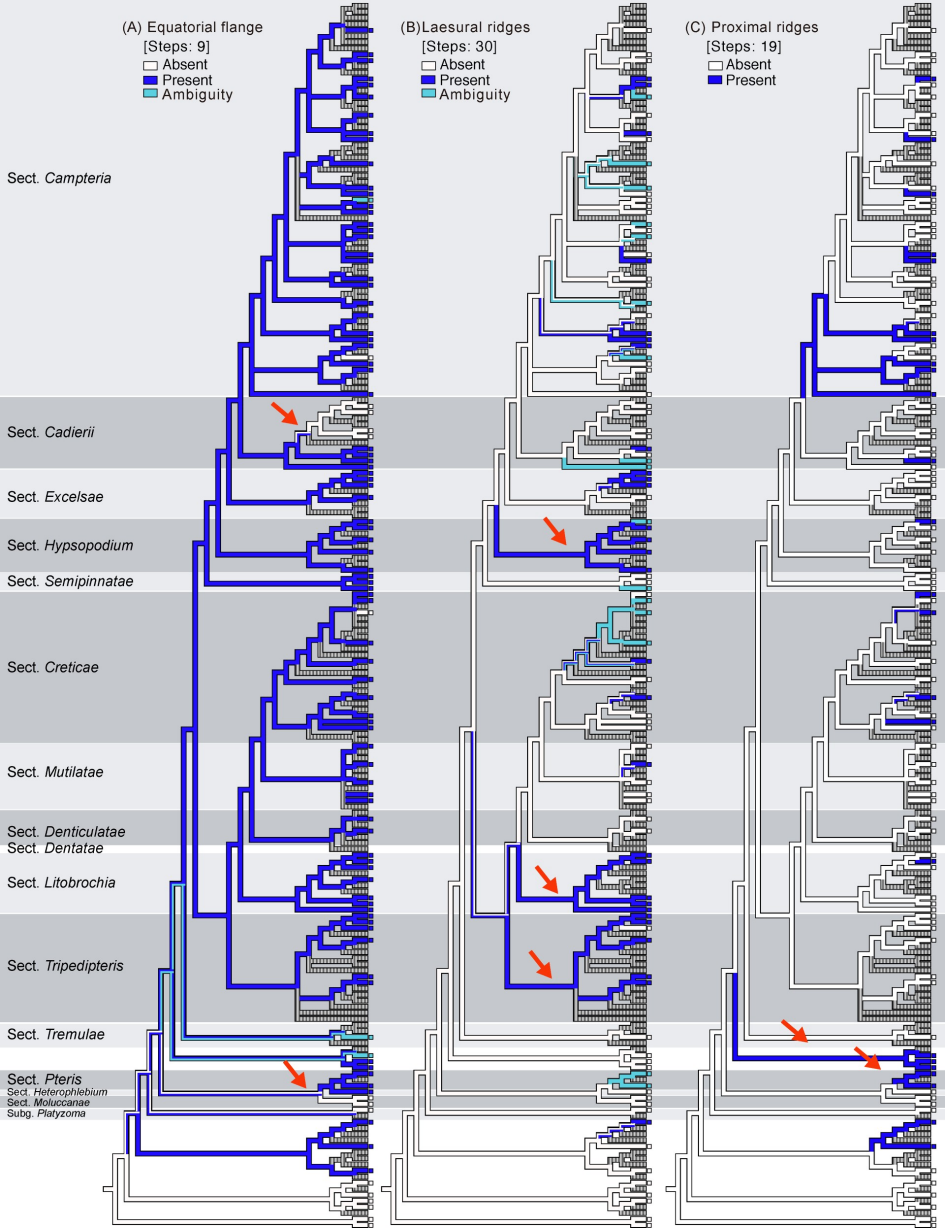
Ancestral character reconstruction for *Pteris* performed by Mesquite. Phylogenetic relationships (BI tree in Figs 1 and 2) of *Pteris* are shown in relation to (A) an equatorial flange, (B) laesural ridges, (C) proximal ridges, (D) distal ridges, (E) tubercula on distal faces, (F) coarse reticula on distal faces, and (G) a row of extervermiculi between the distal face and equatorial flange. Arrows indicated the major clades with the synapomorphic characters. (H) Common spores of each taxa group are mapped.

**Fig 4:**
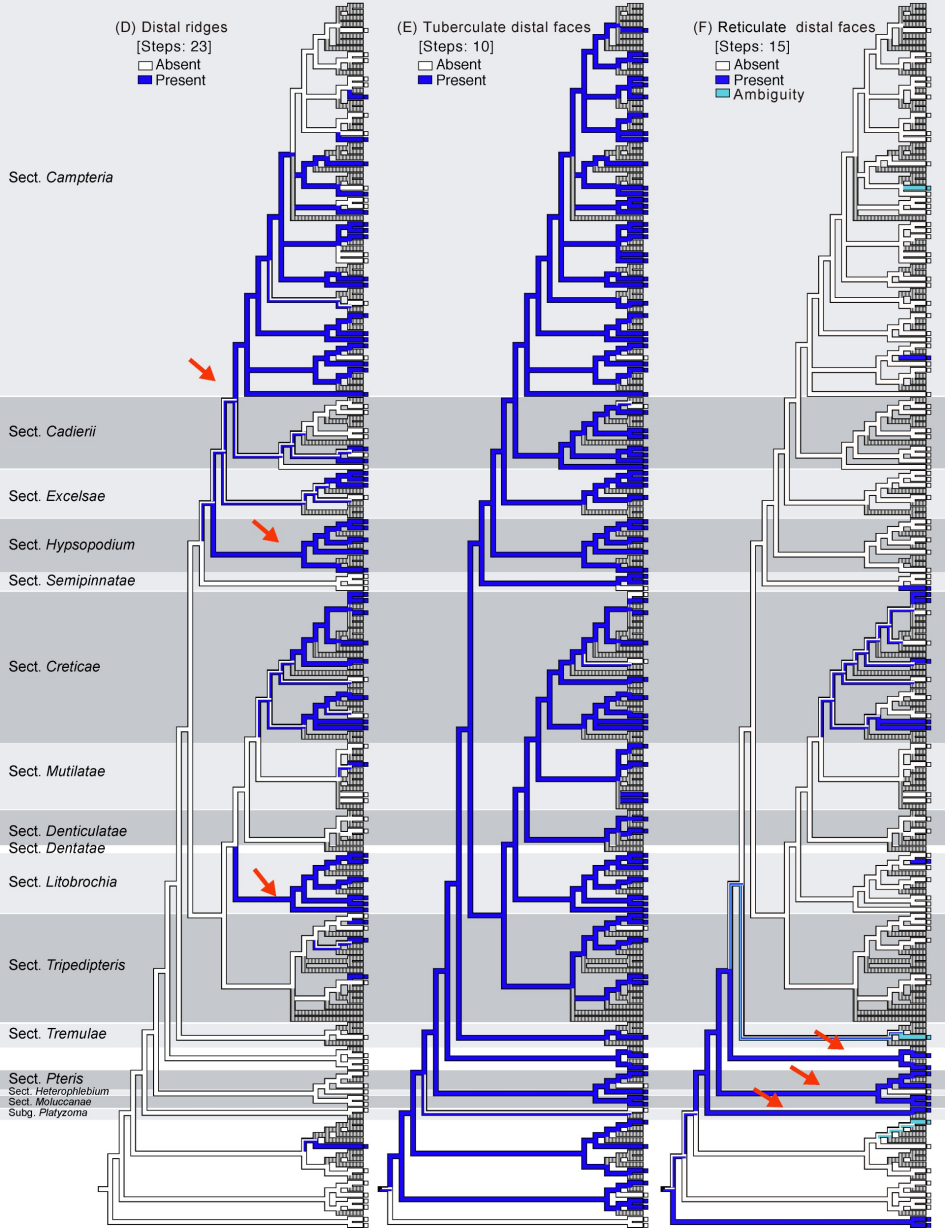
Ancestral character reconstruction for *Pteris* performed by Mesquite (continued).

**Fig 5:**
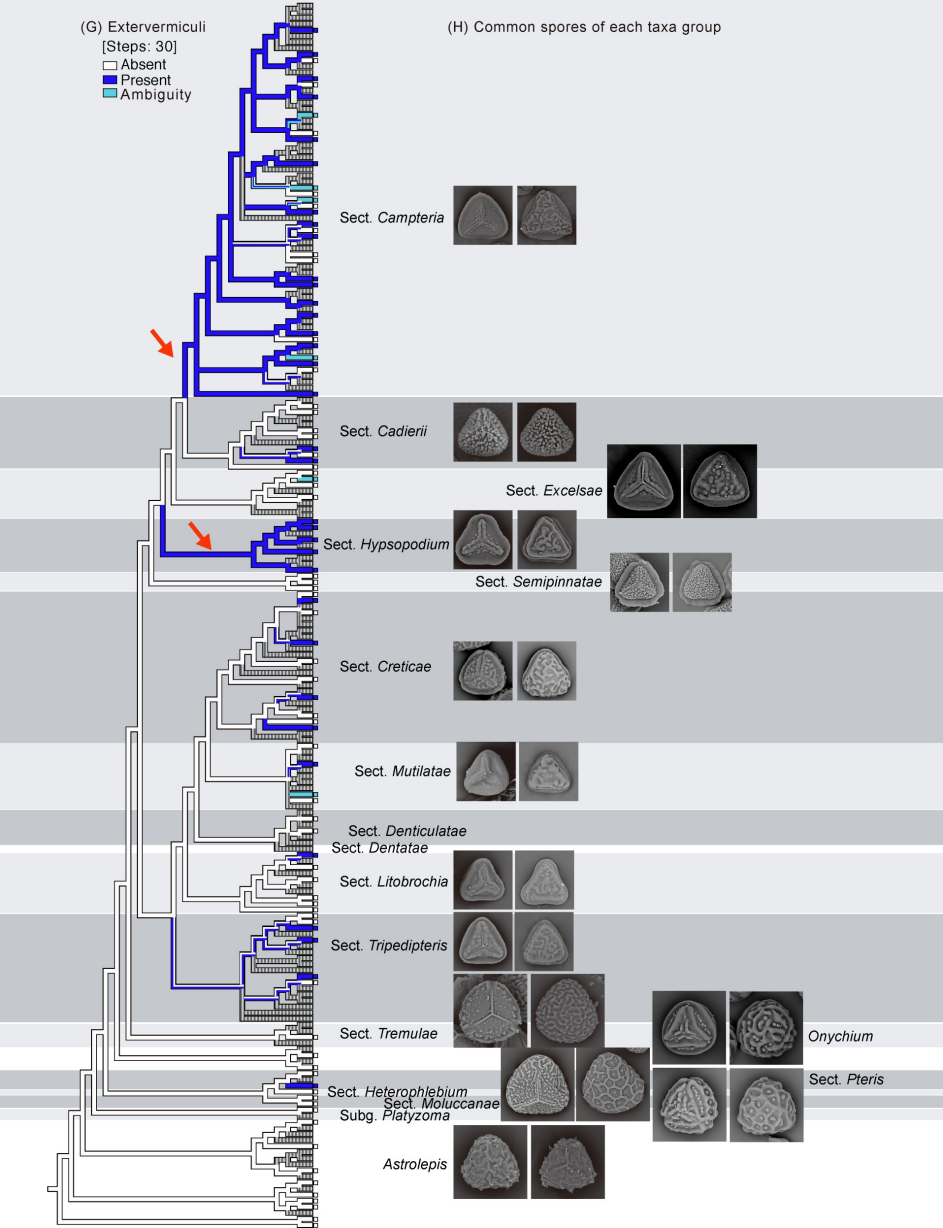
Ancestral character reconstruction for *Pteris* performed by Mesquite (continued).

Phylogenetic analyses (Bayesian inference) were performed based on the combined datasets of *rbc*L and *mat*K markers. The topology obtained was similar to that in previous studies [3]; the 180 *Pteris* species were grouped into three major clades with strong support (BPP = 1; Figs 1 and 2), corresponding to three subgenera, *Campteria*, *Platyzoma*, and *Pteris* [2]. The tree topology further allowed for analysis of spore character evolution (Figs 3–5). However, the relationship implied for the three subgenera was not well supported, and the *Actiniopteris*+*Onychium* clade was included. Subgenus *Campteria* clusters with the *Actiniopteris*+*Onychium* clade first (BPP = 0.54), then with subgenus *Pteris* (BPP = 0.53), and finally with subgenus *Platyzoma* (BPP = 0.6). In the studies of Zhang et al. [1,2], the *Actiniopteris*+*Onychium* clade is the closest taxa of genus *Pteris* (discussed below).

Under subgenus *Campteria* and subgenus *Pteris*, the section circumscription corresponded to the section proposed by Zhang & Zhang [2]. However, the phylogenetic positions of some sections are different, including *Dentatae*, *Denticulatae*, *Litobrochia*, and *Tripedipteris*. The branches connected to the sections were short; some of them had low supported values, both in Fig 2 and Zhang & Zhang’s study [2]. The topology in this study is (((*Creticae*, *Mutilatae*), (*Dentatae*, *Denticulatae*), *Litobrochia*), *Tripedipteris*), different from (((((*Creticae*, *Mutilatae*), *Dentatae*), *Litobrochia*), *Tripedipteris*), *Denticulatae*) of Zhang & Zhang’s study [2]. We discuss this more below.

### Spore morphology and specific character of infrageneric classification

**Fig 6:**
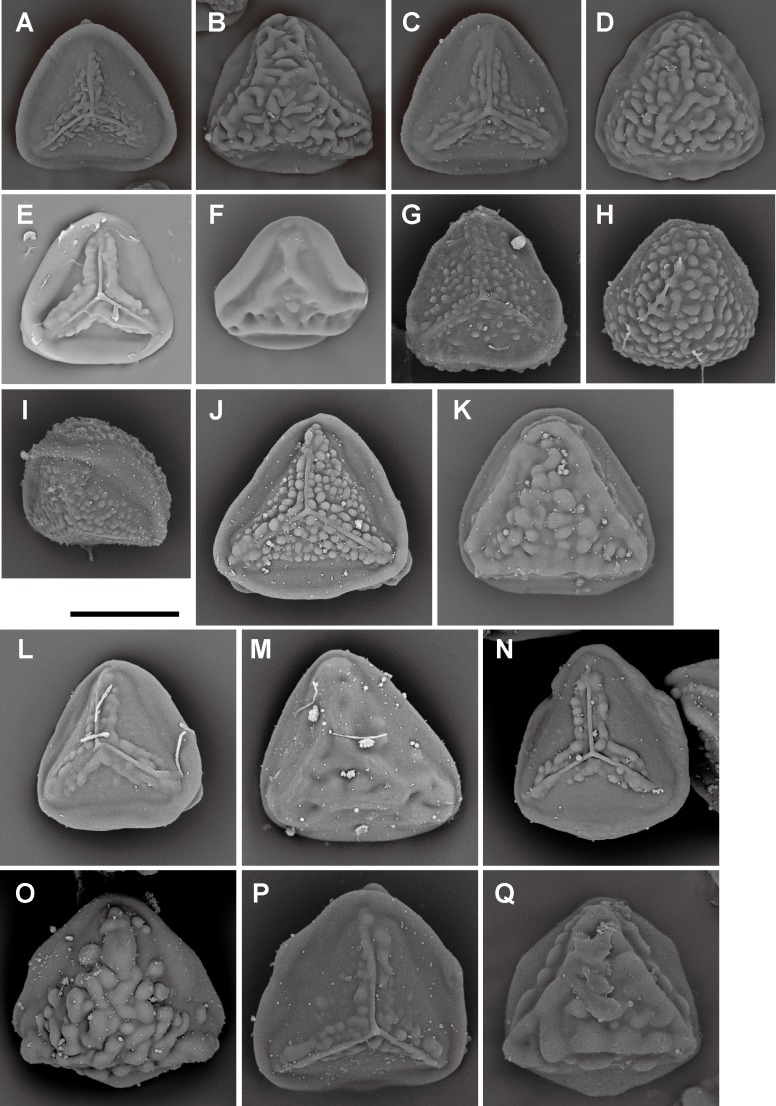
Spore morphologies of *Pteris* section *Campteria* part I. A, B, *P*. *laurisilvicola* Sa.Kurata; C, D, *P*. *oshimensis* Hieron.; E, F, *P*. *giasii* Fraser-Jenk. & Pasha; G, H, I, *P*. *argyraea* T. Moore; J, K, *P*. *aspericaulis* Wall. ex J. Agardh; L, M, *P*. *himalayensis* S.R.Ghosh; N, O, *P*. *setulosocostulata* Hayata; P, Q, *P*. *spinescens* C. Presl. Proximal view: A, C, E, H, J, L, N, P; distal view: B, D, F, I, K, M, O. Scale bars = 30 μm.

**Fig 7:**
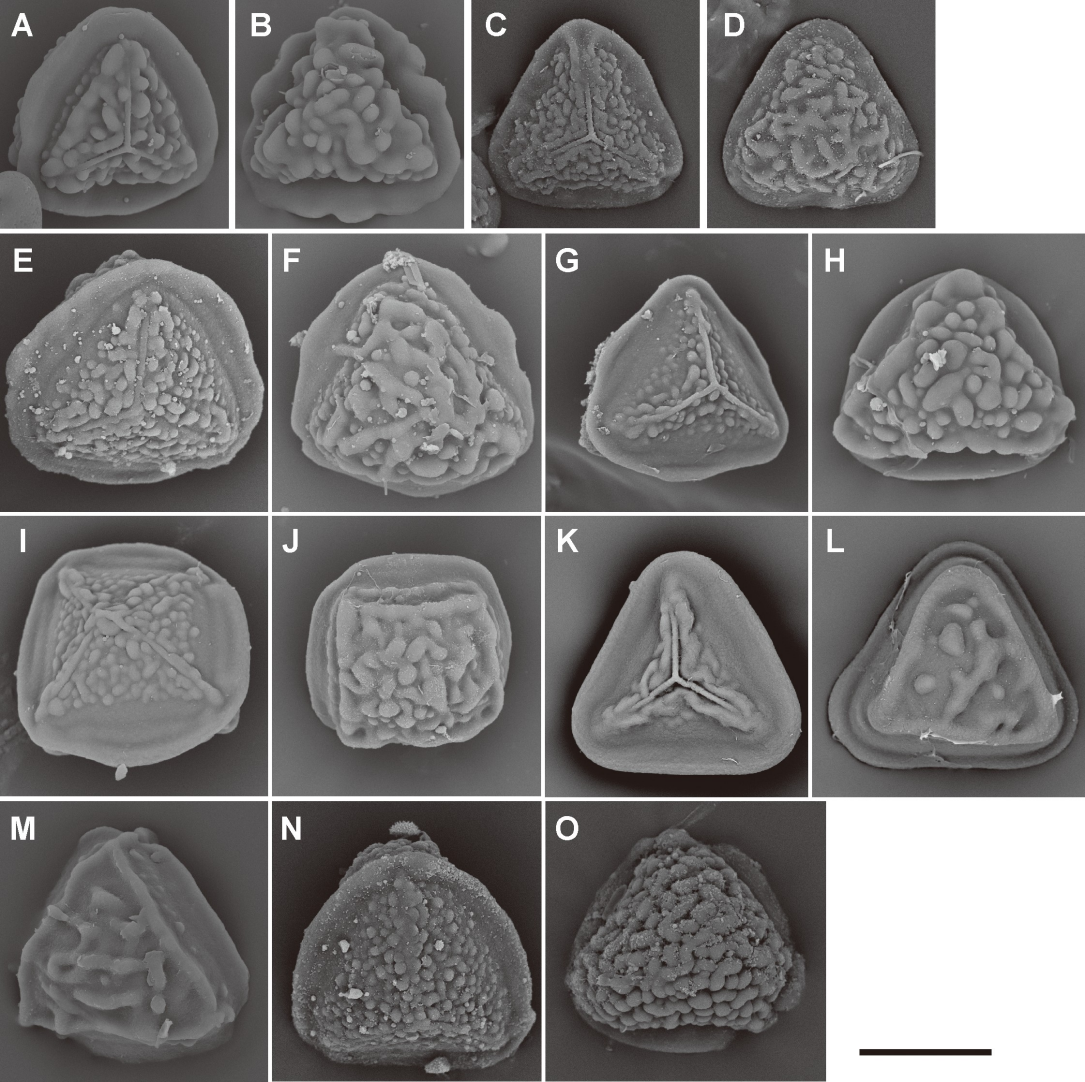
Spore morphologies of *Pteris* section *Campteria* part II. A, B, *P*. *khasiana* (C.B.Clarke) Hieron.; C, D, *P*. *otaria* Bedd.; E, F, *P*. *assamica* Fraser-Jenk. & T.G.Walker; G, H, I, J, P. *biaurita* L. subsp. *Walkeriana* Fraser-Jenk. & Rajkumar; K, L, M, P. *humbertii* C.Chr.; N, O, *P*. *praetermissa* T.G.Walker. Proximal view: A, C, E, G, I, K, N; distal view: B, D, F, H, J, L, M, O. Scale bars = 30 μm.

**Fig 8:**
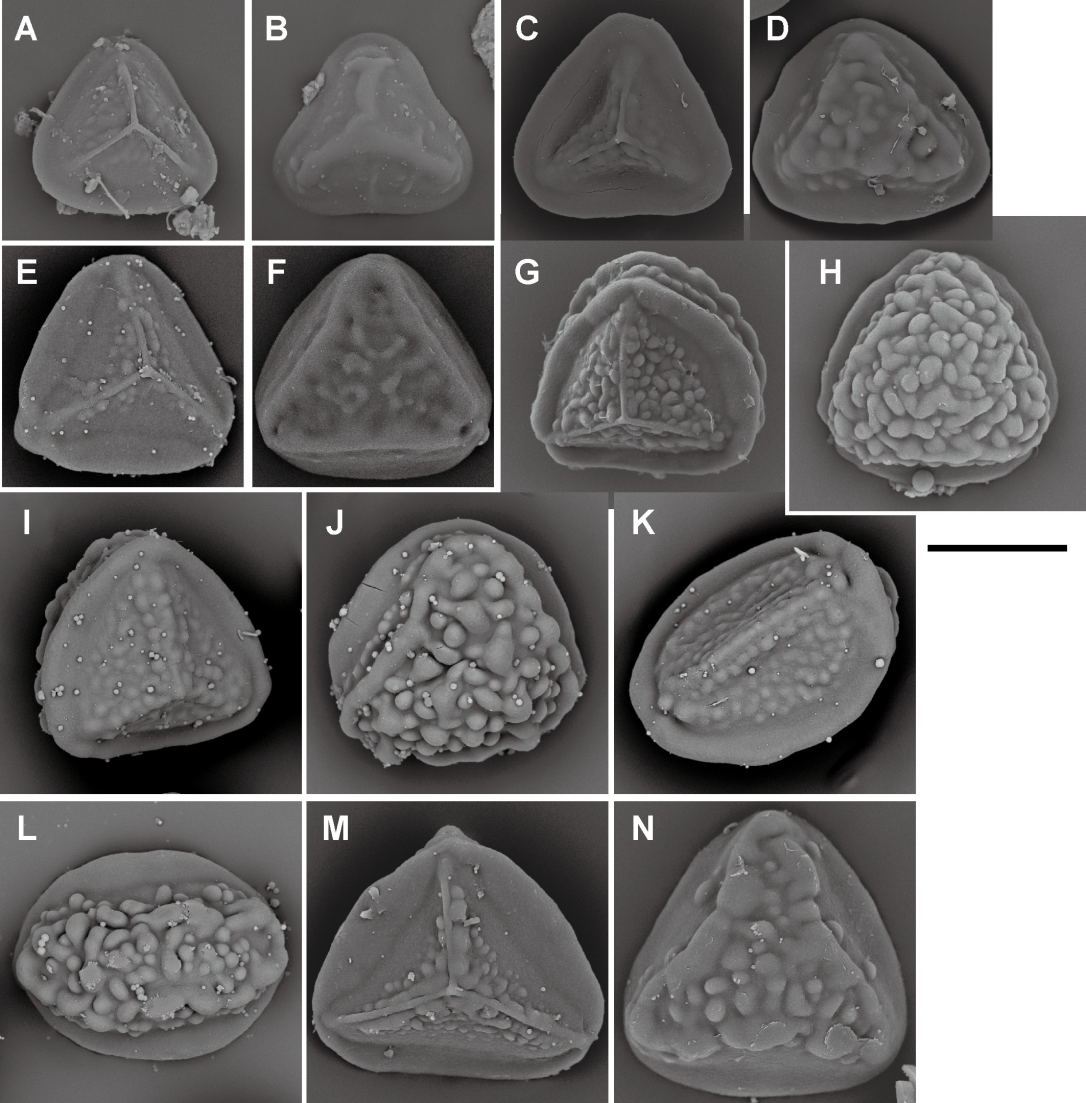
Spore morphologies of *Pteris* section *Campteria* part III. A, B, *P*. *asperula* J.Sm.; C, D, *P*. *longipinnula* Wall. ex J. Agardh; E, F, *P*. *subindivisa* C.B.Clarke; G, H, *P*. *glaucovirens* Linden; I, J, K, L, *P*. *biaurita* L.; M, N, *P*. *roseolilacina* Hieron. Proximal view: A, C, E, G, I, K, M; distal view: B, D, F, H, J, L, M. Scale bars = 30 μm.

**Fig 9:**
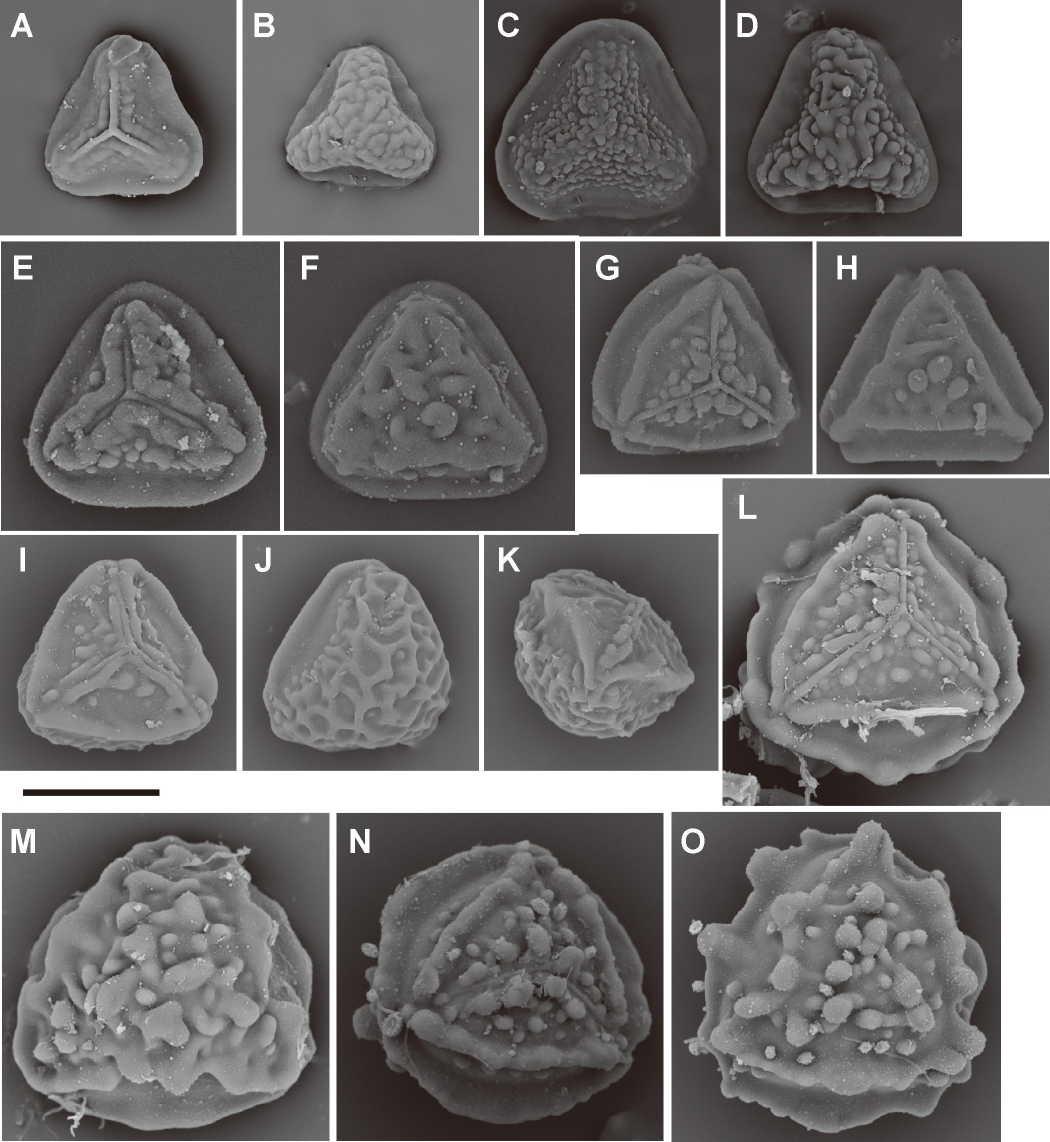
Spore morphologies of Pteris section Campteria part IV. A, B, P. *friesii Hieron*.; C, D, *P. quadriaurita* Retz.; E, F, *P. reptans* T.G.Walker. G, H, *P. subquinata* Wall. ex J. Agardh; I, J, K, P. cf. *mertensioides* Willd.; L, M, *P. normalis* D.Don; N, O, *P. kathmanduensis* Fraser-Jenk. & T.G.Walker. Proximal view: A, C, E, G, I, L, N; distal view: B, D, F, H, J, M, O. Scale bars = 30 mm.

**Fig 10:**
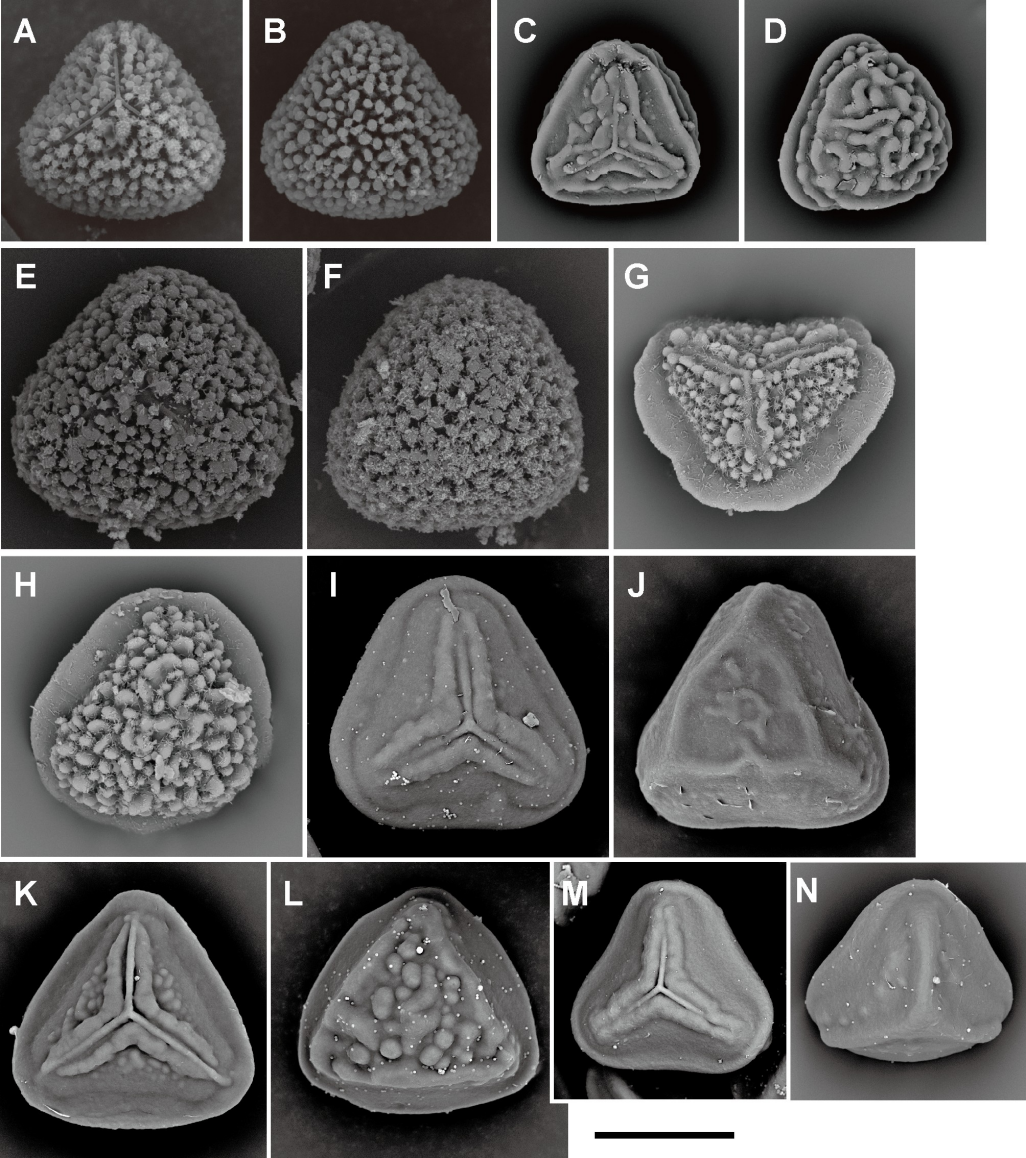
Spore morphologies of *Pteris* section *Cadierii* and section *Excelsae*. A, B, *P*. *grevilleana* Wall. ex J. Agardh; C, D, *P*. *longipinna* Hayata; E, F, *P*. *hainanensis* Ching; G, H, *P*. *venusta* Kunze (section *Cadierii*). I, J, *P*. *formosana* Baker; K, L, *P*. *terminalis* Wall. ex J. Agardh; M, N, *P*. *torricelliana* Christ in K.Schum. & Laut. (section *Excelsae*). Proximal view: A, C, E, G, I, K, M; distal view: B, D, F, H, J, L, N. Scale bars = 30 μm.

**Fig 11:**
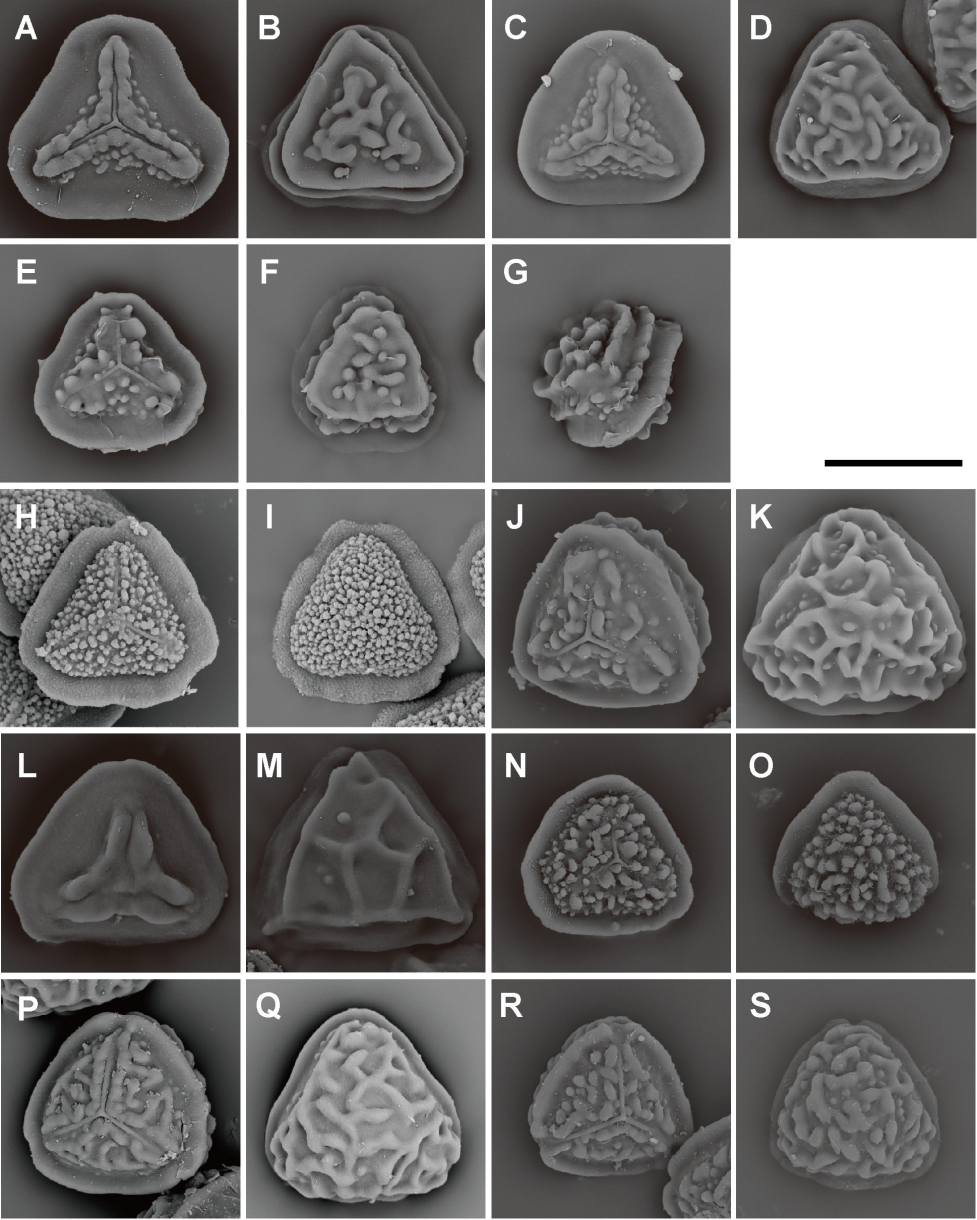
Spore morphologies of *Pteris* section *Hypsopodium*, section *Semipinnatae*, and section *Creticae*. A, B, *P*. *bella* Tagawa; C, D, *P*. *porphyrophlebia* C.Chr. & Ching in Ching; E, F, G, *P*. *pteridioides* (Hook.) F.Ballard (section *Hypsopodium*). H, I, *P*. *dissitifolia* Baker (section *Semipinnatae*). J, K, *P*. *actiniopterioides* Ching; L, M, *P*. *deltodon* Baker; N, O, *P*. *morii* Masam.; P, Q, *P*. *multifida* Poir.; R, S, *P*. *umbrosa* R. Br. (section *Creticae*). Proximal view: A, C, E, H, J, L, N, P, R; distal view: B, D, F, I, K, M, O, Q, S. Scale bars = 30 μm.

**Fig 12:**
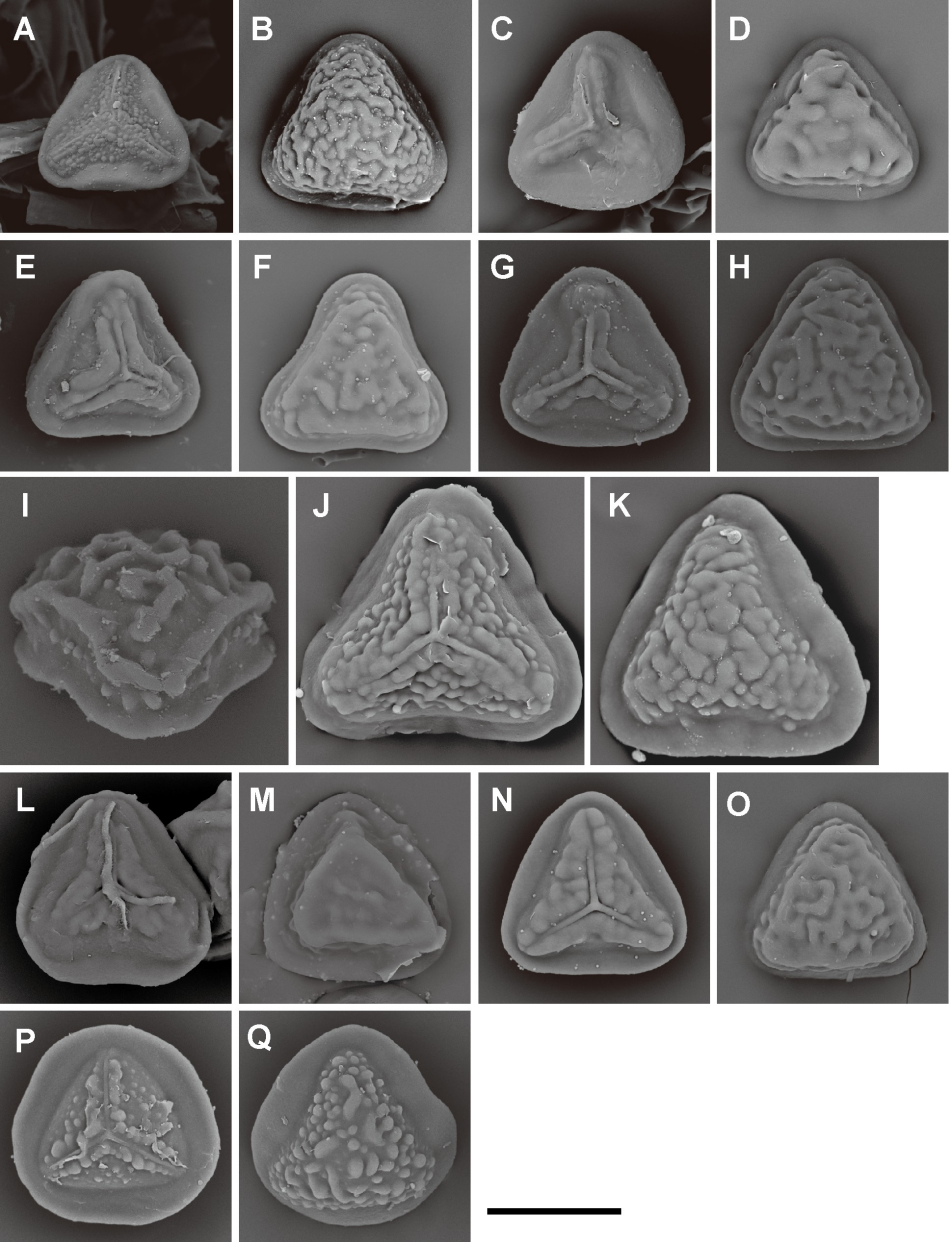
Spore morphologies of *Pteris* sections *Mutilatae*, *Litobrochia*, and *Tripedipteris*. A, B, *P*. *geminata* Wall. ex J. Agardh; C, D, *P*. *griseoviridis* C.Chr. (section *Mutilatae*). E, F, *P*. *altissima* Poir.; G, H, *P*. *podophylla* Sw.; I, *P*. *muricata* Hook. (section *Litobrochia*). J, K, *P*. *macilenta* A.Rich.; L, M, *P*. *macrophylla* Copel.; N, O, *P*. *comans* G. Forst.; P, Q, *P*. *tripartita* Sw. (section *Tripedipteris*). Proximal view: A, C, E, G, J, L, N, P; distal view: B, D, F, H, K, M, O, Q. Scale bars = 30 μm.

**Fig 13:**
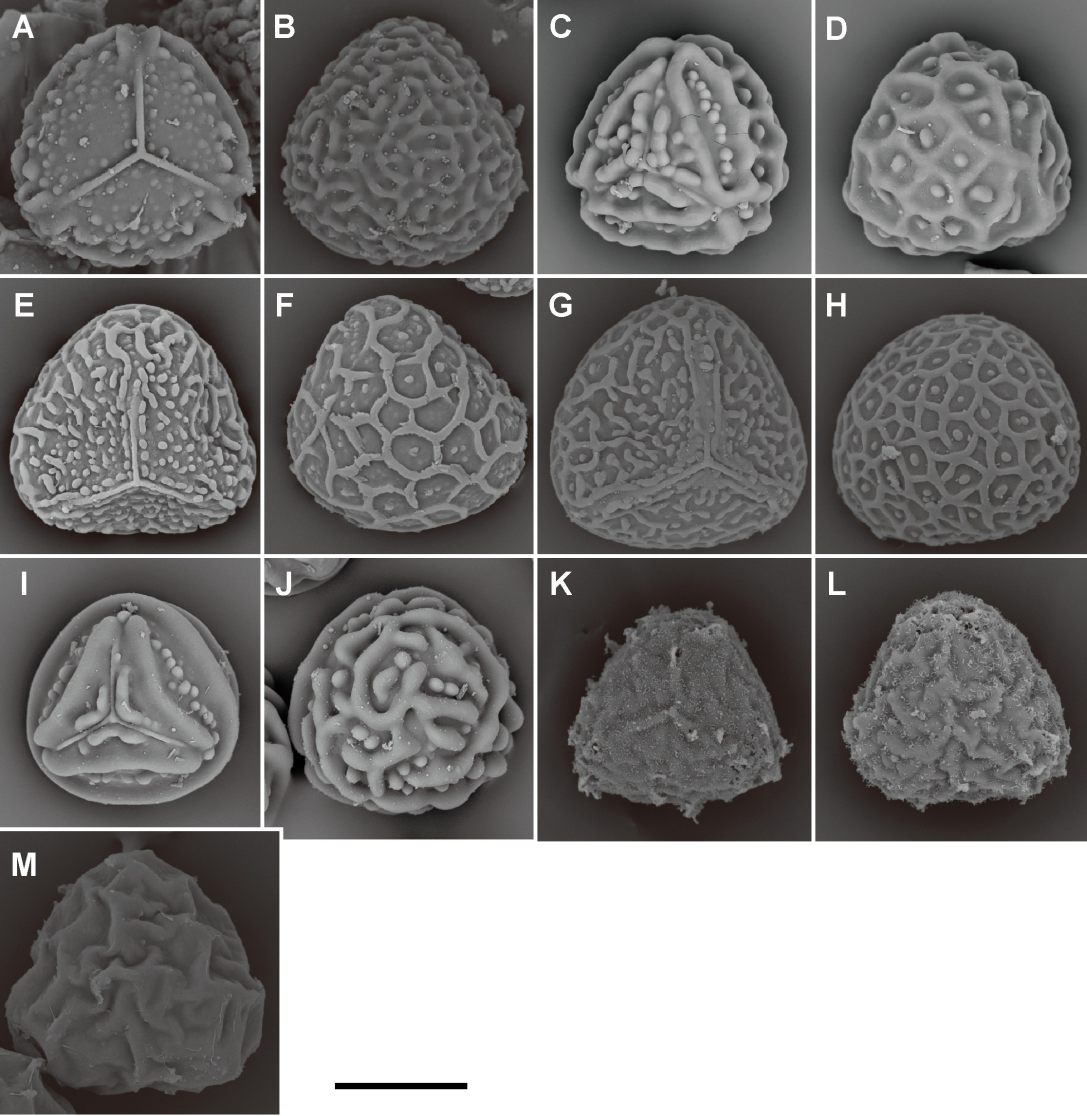
Spore morphologies of *Pteris* sections *Tremulae*, *Pteris*, *Moluccanae*, genus *Onychium*, and genus *Astrolepis*. A, B, *P*. *tremula* R.Br. (section *Tremulae*); C, D, *P*. *longifolia* L. (section *Pteris*), E, F, *P*. *moluccana* Blume, G, H, *P*. *papuana* Ces. (section *Moluccanae*); I, J, *O*. *siliculosum* (Desv.) C.Chr. (genus *Onychium*); K, L, *A*. *cochisensis* (Goodd.) D.M. Benham & Windham, M, *A*. *sinuata* D.M.Benham & Windham (genus *Astrolepis*). Proximal view: A, C, E, G, I, K; distal view: B, D, F, H, J, L, M. Scale bars = 30 μm.

Using SEM, spore morphologies of 57 *Pteris* species, one *Onychium* species, and two *Astrolepis* species were examined; 40 species of them are reported here for the first time. Proximal and distal views of each species were presented (Figs 6–13) based on Zhang & Zhang's infrageneric classification [2]. The section *Campteria* has the most species in this study, and they were divided into four parts for further analysis of spore morphology. Spores of most *Pteris* species are tetrahedral and trilete. The proximal view is usually triangular with rounded corners. Some abnormal spores are found, such as monolete spores of *P*. *biaurita* L. (Fig 8K and 8L), and tetralete spores of *P*. *biaurita* var. *walkeriana* Fraser-Jenk. & Rajkumar (Fig 7I and 7J). In total, the spores of 100 species were analyzed for infrageneric delimitation, including 60 species reported in the present study and 40 species from previous studies (Figs 1 and 2, S1 Table).

Spore morphologies of species within a section are more or less similar but not consistent for all species within a section. A summarized overview of common morphology of each taxon mapped the phylogenetic tree is given in Fig 5H. All the spores of subgenus *Platyzoma*, subgenus *Pteris*, and the section *Tremulae* have no distinct prominent equatorial flange and are relatively rounder than those other taxa. While the ornamentation of proximal and distal faces are different in the sections *Pteris* and *Tremulae*, their equatorial flange can be discerned. Spores of the section *Pteris* have deeper coarse reticula than other spores. Proximal and distal faces are distinctly different in spores of subgenus *Campteria*. Except for section *Tremulae* and some species in section *Cadierii*, the proximal view is triangular with prominent equatorial flanges. Because of the presence of laesural ridges, section *Hypsopodium*, section *Litobrochia*, and section *Tripedipteris* are similar to each other, although those sections arose independently. Comparatively, laesural ridges are absent in certain groups: section *Cadierii*, section *Creticae*, section *Semipinnatae*, and the clade of sections *Mutilatae* and *Dentate*. Moreover, both section *Cadierii* and section *Semipinnatae* have prominent tubercula.

The spore sizes in *Pteris*, based on equatorial diameter, range from 35–65 μm. The smallest spores are about 35–40 μm, such as in *P*. *longipinna* Hayata (Fig 10C and 10D) and *P*. *deltodon* Baker (Fig 11L and 11M). Most of spores are smaller than 60 μm in equatorial diameter. Few species have spores larger than 60 μm in equatorial diameter, only *P*. *normalis* D. Don (Fig 9L and 9M) and *P*. *kathmanduensis* Fraser-Jenk. & T.G. Walker (Fig 9N and 9O).

### Spore character evolution

Seven selected characters were coded (Figs 1 and 2), and ancestral character state evolution combining the phylogeny and the morphology matrix was performed to infer character transitions of *Pteris* spores (Figs 3–5). Of the seven selected characters, none were synapomorphic for *Pteris* with respect to other genera in Pteridaceae. All the characters arose and reversed several times in the different lineages.

Equatorial flanges are plesiomorphic for *Pteris* and show the fewest transitions (nine steps, Fig 3A). Having an equatorial flange is plesiomorphic; few *Pteris* spores have no equatorial flanges. Distinctly different spores were found in section *Cadierii* (*P*. *grevilleana* Wall. ex J. Agardh and *P*. *hainanensis* Ching; Fig 10), section *Moluccanae* (*P*. *moluccana* Blume and *P*. *papuana* Ces.; Fig 13), and subgenus *Platyzoma*. Equatorial flanges also found in the genera in Pteridaceae, including *Anogramma*, *Astrolepis*, *Onychium*, *Pityrogramma*, and *Taenitis* (Fig 13).

Laesural ridges present distinctly in some species. However, many species have ambiguous morphology between laesural ridges or just simple prominences on the proximal face and are difficult to code, i.e., some simple prominences could partially fuse into laesural ridges and independent prominences on proximal faces. Those species were coded as polymorphic. Because of those species with polymorphy, the character of laesural ridges does not seem stable in *Pteris*, and evolved many times (30 steps, Fig 3B). For the species with distinctly laesural ridges, the character is a shared character of species in the section *Hypsopodium* (Fig 11A, 11C and 11E), section *Litobrochia* (Fig 12E and 12G), and section *Tripedipteris* (Fig 12J, 12L, 12N and 12P).

Only the spores with equatorial flanges could have both proximal ridges as well as distal ridges. The species with proximal ridges parallel to the equatorial flange are scattered in different clades (19 steps, Fig 3C). However, the presence of a proximal ridge is a synapomorphy for section *Pteris* (*P*. *longifolia* L., Fig 13C) and the *Actiniopteris*+*Onychium* clade (Fig 13I). A distal ridge can be found in most of the sections in the genus *Pteris* (23 steps, Fig 4D). It is a synapomorphy for section *Campteria*, section *Hypsopodium* (Fig 11B, 11D and 11F), and section *Litobrochia* (Fig 12F, 12H and 12I).

For the ornamentation on distal faces, tubercula are more common than reticula. The presence of tuberculate distal faces is an ancestral state and autapomorphy in several species (10 steps, Fig 4E). Morphologies of tubercula are diverse. Some tubercula on distal faces connect with each other and fuse together, forming vermicular constructions (Fig 6B and 6D). Furthermore, prominently tuberculate digitations are found in several species in different lineages: *P*. *hainanensis* (Fig 10E and 10F), *P*. *grevilleana* (Fig 10A and 10B), and *P*. *venusta* Kunze (Fig 12G and 12H) in section *Cadierii*; *P*. *ensiformis* Burm. f. (clade A3) in section *Excelsae*; *P*. *dissitifolia* Baker in section *Semipinnatae* (Fig 11H and 11I); and *P*. *morii* Masam. (Fig 11N and 11O) in section *Creticae*. Furthermore, the tubercula with rodlets (fascicles rodlets) were only found in section *Cadierii*. This character is similar to that in *Taenitis* species [10], especially *T*. *requiniana* (Gaud.) Copel. *Taenitis requiniana* has no equatorial flange either like *P*. *hainanensis* (Fig 10E and 10F) and *P*. *grevilleana* (Fig 10A and 10B).

The presence of coarse reticula on distal faces is the character most like a synapomorphy in genus *Pteris*, while the most phylogenetically close genera *Actiniopteris* and *Onychium* are included (15 steps, Fig 4F). The characters occur in basal groups, section *Moluccanae*, section *Pteris*, *P*. *platyzomopsis*, and *Actiniopteris*+*Onychium* clade; and are absent in subgenus *Campteria*. The character reverses back to be present in several species, especially in section *Creticae*. Most spores with coarse reticula also have tubercula, including *P*. *actiniopteroides* Ching (Fig 11J and 11K), *P*. *deltodon* (Fig 11L and 11M), *P*. *longifolia* (Fig 13C and 13D), *P*. *moluccana* (Fig 13E and 13F), *P*. *papuana* (Fig 13G and 13H), and *Onychium siliculosum* (Desv.) C.Chr. (Fig 12I and 12J).

The presence or absence of one row of extervermiculi between a distal face and an equatorial flange is difficult to code in some species. It could be varied among different spores within species. For the species with one distinct row of extervermiculi, the character is a synapomorphy for sections *Campteria* and *Hypsopodium* (Fig 11B and 11E).

## Discussion

### Evolution of *Pteris* spore morphology

This study examined spores of most species of *Pteris* and, to our knowledge, is the only one to compare and examine the phylogeny of *Pteris* in terms of phylogenetic patterns of spore morphology. Equatorial flanges and tubercula on distal faces are shared characters of the genus *Pteris*, but are plesiomorphies. Equatorial flanges, tuberculate distal faces, and reticulate distal faces show the fewest transitions among the seven selected characters. Equatorial flanges and tuberculate distal faces (rugate surface) are the primary features for the fossil spores, especially *Polypodaceoisporites*, to be allied with *Pteris* [10, 29]. Our results revealed that the presence of equatorial flanges and tuberculate distal faces are plesiomorphic for *Pteris* and supported the inference about fossil spores.

The presence of laesural ridges, proposed by Palacios-Rios et al. [14], is currently relatively underestimated and synapomorphic in some sections. Most characters change frequently in different lineages, including proximal ridges, distal ridges, tubercula on distal faces, coarse reticula on distal faces, and a row of extervermiculi between the distal face. In bolbitidoid ferns (Dryopteridaceae), the microstructure of perines also reversed several times [8]. It has been reported that similar perine morphologies of different species could have different ontogenies [7]. The resemblances of spores from different species could arise from convergence or parallel evolution, but the factors to determine spore morphological traits have not been revealed [30].

Here, we consider possible environmental factors. In *Pteris*, there is a transition of habitat preference from the terrestrial to lithophytic habitats of section *Cretica* [3], which is more or less consistent with the presence of the coarse reticula on distal faces in this section. Interestingly, several lithophytic *Pteris* species in other sections also possess coarse reticula on distal faces, such as *P*. *longifolia* and *P*. *vittata*. We also compared the geographic distribution [3] with the spore morphology, but no distinct pattern was found. The possible relationship between the coarse reticula on distal faces and lithophytic species needs to be further studied.

### *Pteris* spore morphology for infrageneric classification

The equatorial flanges, reticulate distal faces, and laesural ridges corresponded more to section classification. However, most characters reversed frequently. Mostly, spore morphology is useful at lower taxonomic levels, i.e., species delimitation, rather than section circumscription. Even combining characters did not help characterize most sections of *Pteris*, except sections *Moluccanae* and *Pteris*. Synapomorphies of section *Moluccanae* are coarse reticula on distal face and the absence of an equatorial flange. Synapomorphies of section *Pteris* are proximal ridges and reticulate distal faces. The results in this study showed that the inconsistency of spore combining characters with infrageneric classification reported in a previous study [11] are arisen by multiple origins of spore characters, and not only arisen by the traditionally circumscribed infrageneric classification. In general, each section of *Pteris* has its own specific spore morphology, but species exceptions were found in many sections.

Spore characters, similar to leaf morphologies, reversed several times, but the combination of both characters could be useful. For example, section *Semipinnatae* has pectinate basiscopic pinnae and entire acroscopic pinnae, features that separate it from sections *Cadierii* and *Hypsopodium* [1]. However, the characters can also be found in *P*. *dimorpha* Copel. and *P*. *hainanensis* in section *Cadierii*. If considering the spore characters, the presence of equatorial flanges in section *Semipinnatae* can help differentiate *P*. *dimorpha* and *P*. *hainanensis*, which both lack equatorial flanges.

For the different phylogenetic positions of sections *Dentatae*, *Denticulatae*, *Litobrochia*, and *Tripedipteris*, the evolution of spore characters is considered. The topology is (((*Creticae*, *Mutilatae*), (*Dentatae*, *Denticulatae*), *Litobrochia*), *Tripedipteris*) in this study, but was (((((*Creticae*, *Mutilatae*), *Dentatae*), *Litobrochia*), *Tripedipteris*), *Denticulatae*) in Zhang and Zhang’s study [2]. The spore morphologies of sections *Denticulatae*, *Litobrochia*, and *Tripedipteris* (sections *Dentatae* unsampled in this study) are different in the morphologies of their laesural (Fig 3B) and distal ridges (Fig 4D). Spores of section *Denticulatae* have no laesural ridges or distal ridges (Fig 2) and are more similar to sections *Mutilatae*. If section *Denticulatae* is the most basal one among the three sections, as in the previous tree [2], the evolutionary transitions of laesural ridges will increase, and the transitions of distal ridges will be constant.

### Spore morphology of *Pteris* and related genera

In this study, the relationships of subgenera *Campteria*, *Platyzoma*, and *Pteris* were not well resolved, including separation of the *Actiniopteris*+*Onychium* clade. The relationship of *Pteris*, *Onychium*, and *Actiniopteris* is different from that in the study by Zhang et al. [1,2]; the genus *Pteris* is monophyletic. *Onychium* and *Actiniopteris* are thought to be the closest taxa to the genus *Pteris* [2,3]. Comparing spore morphologies of *P*. *platyzomopsis* (*Platyzoma microphyllum* in [10,31]), *Onychium* and *Actiniopteris* are more similar to *Pteris* species, especially *P*. *longifolia* and *P*. *vittata*. *P*. *platyzomopsis* is more similar to *Ceratopteris richardii* [10].

Most of the taxa in the genus *Pteris* are diploids, triploids, or tetraploids; there are few pentaploids and hexaploids [32]. The spore sizes are similar to that in a previous study of the genus *Pteris* [10]. Comparing the spore sizes and ploidy levels of some species, the ploidies of different species could be inferred. It is found that sexually diploid species, such as *P*. *longipinna* and *P*. *deltodon*, have spore sizes of 35–40 μm. However, sexual tetraploids, such as *P*. *multifida* and *P*. *umbrosa* R. Br., have similar spore sizes to sexual diploids. Furthermore, apogamous species have varied spore sizes, and there is no clear correlation with ploidy levels. Species with 40–50 μm spores could be apogamous diploid, such as *P*. *laurisilvicola* and *P*. *oshimensis*, or apogamous triploid, such as *P*. *actiniopteroides* and *P*. *incurvata* Y.S. Chao, H.Y.Liu & W.L.Chiou [33]. The correlation among spore sizes, ploidy levels, and reproductive traits is unclear.

## Conclusions

For spores of *Pteris*, the results of this study not only report the morphology but also clarify the use of spore characters in systematic studies of *Pteris*. Spore morphology can circumscribe some sections, with some exceptions. However, because all of the characters occur with reversals, specific synapomorphies are difficult to discern. The application of spore morphology for section circumscription is limited.

## Supporting information

S1 Table Voucher specimens of spore samples examined for this study and GenBank accession numbers for DNA sequences used for the phylogenetic tree construction in this study. The information presented here includes species, spore morphologies, GenBank numbers for *rbcL*, and *matK*, voucher, herbarium, and if the spore sample and DNA sequences are from the same voucher specimen. Spore morphologies from previously published research were recorded and cited.
(XLSX)
